# The transcriptional mechanism behind *Mimosa pudica* leaf folding in response to mechanical disturbance

**DOI:** 10.1007/s00425-025-04830-x

**Published:** 2025-10-06

**Authors:** Matteo Buti, Alice Checcucci, Chiara Vergata, Luciana Renna, Susanna Pollastri, Francesco Loreto, Stefano Mancuso, Federico Martinelli

**Affiliations:** 1 Department of Agriculture, Food, Environmental and Forestry Sciences, University of Florence, Florence, Italy; 2 Department of Biology, University of Florence, Florence, Italy; 3 Institute of Sustainable Plant Protection, IPSP, National Research Council, Sesto Fiorentino (FI), Italy; 4 Department of Biology, University of Naples Federico II, Naples, Italy

**Keywords:** Leaf folding, Mechanical disturbances, Memory acquisition, *Mimosa pudica*, Transcriptome, Transcriptional mechanism

## Abstract

**Main conclusions:**

Repeated stress in *Mimosa pudica *reduces photosystem efficiency, alters gene expression, shifting from flavonoid biosynthesis to stress resistance pathways, offering insights for sustainable plant stress defense strategies.

**Abstract:**

*Mimosa pudica* is a plant known for its ability to fold leaves in response to mechanical disturbances, which serves as a visible phenotypic stress marker. Leaf folding response occurs with a timing and an intensity that vary depending on the stimulus. This adaptive behavior may function as a defense mechanism, helping plant resist herbivores and environmental stressors. In this study, we investigated the gene regulatory networks underlying *M. pudica* leaf closure following single and multiple mechanical disturbances (whole pot drops). Chlorophyll fluorescence was measured as fast phenotypic indicator of transient or permanent photochemical damage, and transcriptional responses were measured to identify the key genes regulating phenotypic changes after single or multiple drops. A progressive reduction of the quantum yield of PSII revealed a lower electron transport rate in leaves subjected to one or more drops, which may indicate the onset of energy shortage, potentially caused by limited ATP availability that constrains both leaf movement and photosynthesis. The transcriptomic profiles revealed larger differences when plants were subjected to multiple drops than to a single drop, with respect to unstressed controls. Interestingly, following a single drop, the majority of up-regulated genes were associated with the flavonoid biosynthetic pathway. After multiple drops, however, genes associated with biotic and abiotic stress resistance pathways were predominantly up-regulated. These findings provide new insights into the gene regulatory networks driving stress-induced movements in *M. pudica* leaves and lay the groundwork for developing sustainable strategies for plant stress defense.

**Supplementary Information:**

The online version contains supplementary material available at 10.1007/s00425-025-04830-x.

## Introduction

To adapt to their sessile nature, plants have evolved growth responses that enable them to cope with environmental changes (Hagihara and Toyota 2020). A key strategy involves a physiological state of adaptive preparedness that enables plants to respond to stress faster and more efficiently (Harris et al. 2023). Several studies have shown that plants exhibit mnemonic functions regulating specific physiological and morphological responses, often mediated by genetic or epigenetic mechanisms (He and Li 2018). Understanding the molecular basis of this alert state, including the initial perception of stimuli, rapid response, and memory of past stress, is particularly relevant (Gagliano et al. 2014).

Mechanical stimulation, such as touch, alters plant growth and development via thigmomorphogenesis (Chehab et al. 2009). Touch triggers changes in intracellular calcium, reactive oxygen species, and several hormones, such as ethylene, jasmonates, abscisic acid, brassinosteroids, and auxin, which activate genes involved in cell wall remodeling, calcium sensing, and defense (Chehab et al. 2009). Among these, *TCH* genes (Monshausen and Haswell 2013) and mechanosensitive ion channels like Piezo play crucial roles in stimulus perception and the consequent signal transduction through regulating calcium ion flow (Kaur et al. 2023).

Genes involved in responses to biotic and abiotic stresses, such as *NPR1* (Nonexpressor of Pathogenesis-Related Genes 1) (Ronga et al. 2015; Zavaliev and Dong 2024), and transcription factors from the WRKY (Meraj et al. 2020), AP2/ERF (Wang et al. 2023), and DREB2 families, have also been implicated in mechanical stress and stress memory mechanisms. Similarly, genes involved in the phenylpropanoid and flavonoid pathways contribute to stress responses through antioxidant activity (Sharma et al. 2019). Finally, DNA methylation and histone modification genes have been found to play a role in plant mnemonic processes, influencing gene expression and chromatin structure underlying memory mechanisms (Liu and He 2020).

Transcriptomic studies in *Arabidopsis thaliana* (Lee et al. 2005) and *Populus nigra* (Fluch et al. 2008) demonstrated that genes that change their expression following single mechanical stimulation mostly involve defense-related mechanisms, such as reactive oxygen species (ROS) production, enzymatic ROS scavenging, and activation of the phenylpropanoid pathway. Two additional studies showed that, following a second stress event, the genes that altered their expression differed from those that responded to a single mechanical stimulus, suggesting a form of desensitization or reprogramming (Pomiès et al. 2017). Short-term transcriptomic responses rapidly activated abiotic stress pathways and plant defense mechanisms, such as ethylene and jasmonic acid signaling, as well as photosynthesis regulation, while later responses impacted genes related to cell wall formation and wood development.

*Mimosa pudica* is a classical model for studying touch-induced leaf folding (Bakshi et al. 2023). Interestingly, leaf folding seems to be sustained longer under high-light conditions compared to light-limited conditions (Jensen et al. 2011). In *M. pudica*, touch-induced leaf closure can also trigger a signal that spreads to nearby leaves, causing even those that were not touched to close (Malone 1994). Although recent studies have begun to explore the role of ion channels and signaling pathways in *M. pudica* leaf movement (Lee et al. 2005; Tran et al. 2021; Hagihara et al. 2022), the gene expression patterns associated with the repeated leaf folding response of *M. pudica* and the underlying biochemical mechanisms remain largely unexplored.

In this study, the transcriptional and physiological responses underlying *M. pudica* leaf closure in response to single and multiple mechanical stimuli were investigated. By comparing plants subjected to single or multiple mechanical stimuli to undisturbed controls, with subsequent reopening of the leaves, we identify the key regulatory genes and pathways related to acclimation and mechanical stress memory. We hypothesize that distinct molecular and genetic mechanisms, still not totally understood, underlie leaf folding responses to single versus multiple mechanical stimuli. This represents the first transcriptomic analysis focused on repeated touch responses in *M. pudica*, advancing our understanding of mechanosensory adaptation in plants.

## Materials and methods

### Experimental set-up and design

Twenty-four plants at identical developmental stages (10 cm tall, 5 leaves brunches), 18-month-old, were purchased from the local specialized nursery Vivaio Calicanto in Florence and grown for a week for acclimatation in a growth chamber with a temperature of 23 °C, a 16/8 h light/dark regime and 60% humidity. Three different treatments were applied to eight plants each. ‘Control’ plants were left non-stimulated, with their leaves wide open. ‘Single-stimulated’ plants were subjected to a single drop from a fixed height. Our experimental apparatus was engineered to deliver a precise and repeatable mechanical stimulus to potted plants. It featured a plastic container, fitted with adjustable hanging mechanisms, which slid along a graduated steel track. This track was firmly anchored to a soft, foam base. Each plant, securely nestled within its container, was manually elevated to a precise height of 15 cm. Upon release, the container and plant descended along the track, impacting the foam base. A specially designed, shallow depression in the foam at the point of impact was crucial; it completely absorbed the kinetic energy, eliminating any bounce. This meticulous design ensured that every plant received an identical physical disturbance, consistently triggering the complete closure of their leaves. The procedure was described by Gagliano et al. (2014). ‘Multi-stimulated’ plants were subjected to repetitive drops from the same height, using the same methodology, with cycles of leaf closing and reopening until the leaves no longer responded to the stimulus (adapted leaves).

Plants subjected to the three different treatments were either measured for chlorophyll fluorescence parameters or sampled for transcriptomic analysis. Control samples were collected quickly cutting the leaf at the hypocotyls base and immediately dropping it in liquid nitrogen to avoid any mechanical stimulation. Samples subjected to a single stimulation were collected from the drop (within seconds) and instantly submerged in liquid nitrogen, while samples subjected to repetitive drops in the case of multiple stimulations were harvested and stored in liquid nitrogen approximately 10 min from the start of the treatment.

### Chlorophyll fluorescence

The Imaging Pam M-series fluorimeter (Walz, Effeltrich, Germany) was used to measure chlorophyll fluorescence parameters in leaves. After the treatment, ‘control’, ‘single-stimulated’ and ‘multi-stimulated’ plants were first adapted to darkness for 20 min, and then individually placed inside the fluorimeter chamber, and PSII maximum quantum yield in leaves was measured as the ratio between variable fluorescence and maximal fluorescence (*F*_v_/*F*_m_). Plants were then exposed to actinic light (110 μmol photons m^−2^ s^−1^) and, after 10 min, the PSII quantum yield in illuminated leaves (Φ PSII) was also measured according to Genty et al. (1989). For each treatment, measurements were performed on five biological replicates.

### RNA isolation and sequencing

Total RNA isolation was performed using approximately 50 mg of the immediately after stimulation frozen material ground with a pestle and mortar following the Norgen Plant/Fungi Total RNA Purification Kit protocol (Norgen, Thorold, Ontario, Canada) with slight modifications in the cell lysis phase. Briefly, proteinase K was added to the lysis buffer C and β-mercaptoethanol mixture, and pulverized samples were heated at 56 °C for 10 min under bead beating condition. For the final elutions, the solutions were brought to 60 μL using RNase-free water. Maximal 10 μg of extracted RNA was treated with DNAase I (NEB) before quality check. RNA integrity was assessed for each sample using the Agilent 2100 Bioanalyzer with the RNA 6000 nano kit (BioRad Inc.).

Sequencing libraries were obtained following the Stranded mRNA Library Prep procedure (Illumina) with an exclusive unique dual index combination (three libraries for each of the three experimental conditions). Qubit™ 4 Fluorometer (dsDNA High Sensitivity Kit; Invitrogen) was used to estimate the concentration of the libraries, while quality assessment was performed by Agilent 2100 Bioanalyzer with DNA HS kit (BioRad Inc.). Novaseq 6000 Reagent Kit (2 × 100 + 10 + 10 cycles) was used to perform sequencing, with all the samples processed in a single flow cell.

### De novo assembly of the *Mimosa pudica* transcriptome

The RNA-Seq obtained from the ‘control’, ‘single-stimulated’ and ‘multi-stimulated’ plants was used to generate a de novo transcriptome assembly of *Mimosa pudica*. After the conversion of RNA-Seq data to fastq files with Illumina bcl2fastq v2.20, RNA-Seq raw reads quality was assessed with FastQC v0.11.5 (Andrews 2010), and adapters sequences and low-quality reads were removed with Trimmomatic v0.39 (Bolger et al. 2014) using these settings: ILLUMINACLIP:adapt.fa:2:30:10 HEADCROP:1 LEADING:3 TRAILING:3 SLIDINGWINDOW:4:18 MINLEN:40.

The RNA reads of all the libraries were used for a de novo assembling with Trinity v2.13.2 (Grabherr et al. 2011), and assembled transcripts were clustered using CD-hit v4.8.1 (Fu et al. 2012) to produce a set of ‘non-redundant’ transcripts. Statistics of non-clustered and clustered de novo assembled transcriptome were obtained with ‘n50’ Perl script from the SeqFu suite (Telatin et al. 2021). The final transcriptome completeness was assessed with BUSCO v5.3.2 (Simão et al. 2015) using the ‘viridiplantae_odb10’ lineage.

### Transcripts level estimation and differential expression analyses

Bowtie2 v2.4.4 (Langmead and Salzberg 2012) with default parameters was used to map back the reads of the sequenced RNA libraries were to the final *Mimosa* transcriptome, while the expression quantification of each transcript was carried out with the Salmon v1.4 (Patro et al. 2017) tools ‘quant’ and ‘quantmerge’.

Raw counts data elaborations and differential expression (DE) analyses were performed with Bioconductor EdgeR v3.38.1 (Robinson et al. 2009). This tool was used to filter out the not active transcripts (a transcript was considered ‘active’ if reads per million mapping was > 1 in two or more libraries), normalize the RNA libraries according to their depth, visualize the multi-dimensional scaling (MDS) plot for RNA libraries normalized counts, and do the differential expression analyses. A transcript was considered as differentially expressed in a pairwise comparison if its false discovery rate (FDR) was lower than 0.05 and its log2 fold change (LFC) was lower than − 2 or higher than 2. Venn diagram representations of differentially expressed transcripts (DETs) were visualized with Venny 2.1.0 (Oliveros 2015) and InteractiVenn (Heberle et al. 2015).

### Functional annotation and enrichment analyses

Functional annotation of DETs were performed on the Galaxy platform (Afgan et al. 2018) using the BLAST + blastx-fast algorithm (Camacho et al. 2009). Searches were conducted against the NCBI nr, SwissProt, RefSeq, TrEMBL, UniRef100 Viridiplantae, and Araport11 protein databases, applying an *e* value cutoff of 10^–3^. To perform enrichment analyses, Gene Ontology (GO) terms were assigned to the entire assembled transcriptome of *M. pudica* using EggNOG (Cantalapiedra et al. 2021) with ‘Viridiplantae’ taxonomic scope. Additionally, KEGG orthologs were mapped using the (KEGG Automatic Annotation Server) (Moriya et al. 2007) with BBH assignment method, using Arabidopsis and available Fabaceae species as reference organisms.

GO enrichment analyses was conducted in Cytoscape 3.10.1 (Shannon et al. 2003) using the BinGO 3.0.5 plugin (Maere et al. 2005), with a hypergeometric test, FDR correction, a significance level of 0.05, and ‘GOSlim_Plants’ ontology. Enriched KEGG classes across the three pairwise comparisons were identified using clusterProfiler v4.6.2 (Wu et al. 2021), via a custom R script. KEGG classes were considered enriched in a comparison if the adjusted *P* value was below 0.05, and the results were visualized using ggplot2 v3.5.1 (Wickham 2016).

## Results

### Response of *Mimosa pudica* plants to dropping

#### Leaves movement

Once subjected to dropping as described by Gagliano et al. (2014), ‘single-stimulated’ plants performed a folding movement of their leaflet within 30 s, soon after also actuating a dropping movement of the petiole (directly attached to the stem). The plant’s entire response to the dropping was usually completed within 1 min. ‘Multi-stimulated’ plants did not actuate any of the movements made by ‘single-stimulated’ plants.

#### Chlorophyll fluorescence measurements

**Fig. 1:**
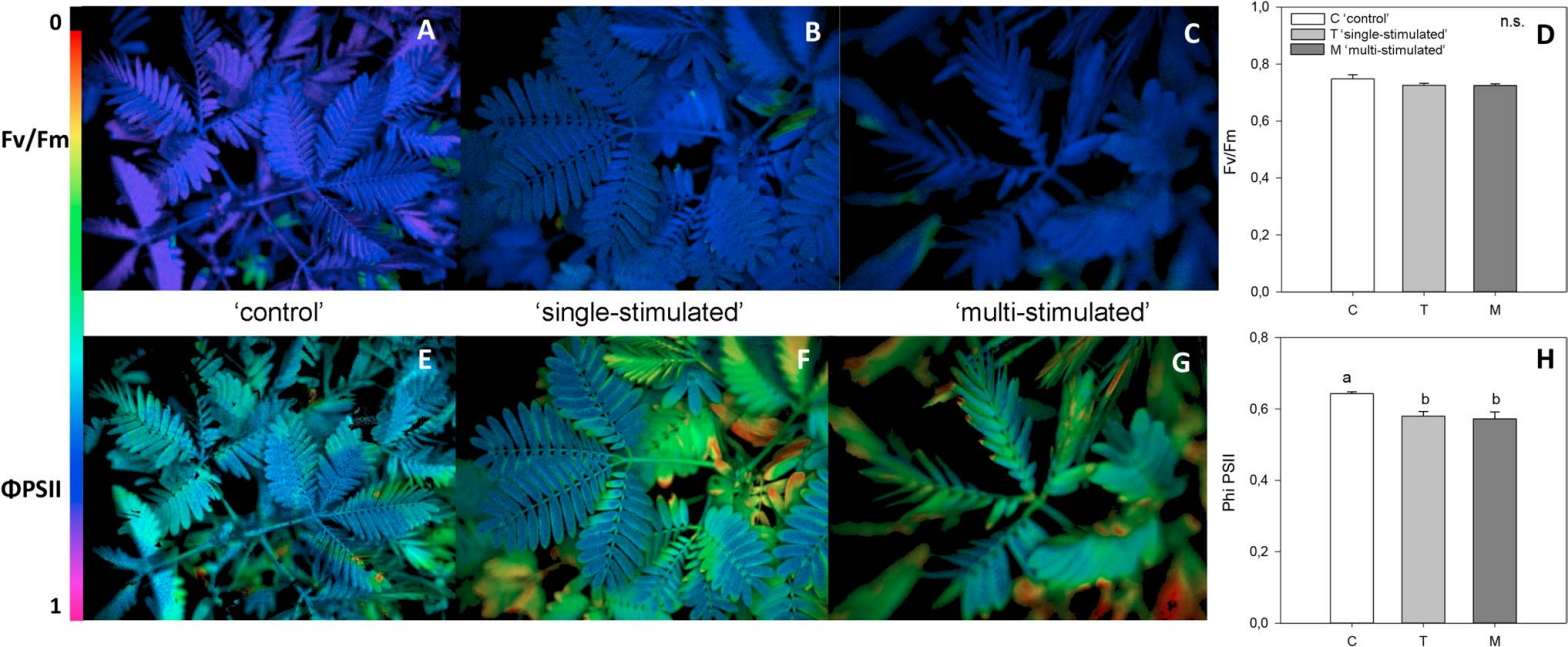
**A**–**H** Representative chlorophyll fluorescence images of PSII maximum quantum yield (*F*_V_/*F*_M_) and PSII quantum yield (*Φ* PSII) in *Mimosa pudica* plants, in ‘control’ (C) (**A**, **E**), ‘single-stimulated’ (T) (**B**, **F**), and ‘multi-stimulated’ conditions (M) (**C**, **G**). Light adapted plants were subjected to ‘control’, ‘single-stimulated’ or ‘multi-stimulated’ treatment and then, after 20 min in the dark, *F*_V_/*F*_M_ measurements were performed. Then, plants were exposed to actinic light (110 PAR), and after 10 min, *Φ* PSII was measured. **D**, **H** The color bar panels showed means ± SE (*n* = 5) of *F*_V_/*F*_M_ and PhiPSII in control conditions (C, white bars), after 1 drop (T, light gray bars), and after multidrop (M, gray bars). A one-way ANOVA followed by Tukey’s test was performed to define statistically significant differences among means (*P* < 0.05). Means not sharing the same letters are statistically significantly different (n.s. not significant). The color bar on the left of the panel shows the ranges of chlorophyll fluorescence

To evaluate the photosynthetic performances of stressed plants, the quantum yield of PSII in leaves of ‘control’, ‘single-stimulated’ and ‘multi-stimulated’ plants *Mimosa* plants was measured by chlorophyll fluorescence (Fig. 1). The maximal quantum yield of PSII in dark-adapted leaves was similar in all the samples (Fig. 1A–D), whereas, in illuminated plants, the PSII quantum yield was significantly higher in ‘control’ than in ‘single-stimulated’ and ‘multi-stimulated’ plants (Fig. 1E–H).

#### RNA sequencing and transcriptome assembling

RNA libraries were sequenced, and the number of resulting paired reads was 120.2 million, ranging from 11.3 to 20.5 million for each library (Table 1). Due to the low sequencing quality of the C3 library, one ‘control’ sample was excluded from the downstream bioinformatic analyses. Once their quality was assessed, the RNA raw reads were deposited with the E-MTAB-14230 accession number in the ArrayExpress database. The percentage of ‘survived’ reads after the elimination of low-quality reads and adapters sequences ranged from 89 to 94% across the libraries (Table 1).

**Table 1:** RNA-seq data analysis. For each library, the number of raw reads, number and percentage of filtered reads, and number and percentage of reads mapped to the de novo assembled *M. pudica* transcriptome were reported

Sample properties			Filtering		Mapping
Treatment	Sample ID	Input read pairs	N° pairs survived	% pairs survived	% aligned pairs
‘Control’	C1	13,703,838	12,701,613	92.69%	90.90%
	C2	20,467,660	18,807,858	91.89%	91.97%
‘Single-stimulated’	T1	17,913,579	16,543,531	92.35%	93.68%
	T2	10,287,641	9,447,847	91.84%	93.54%
	T3	19,158,762	17,455,579	91.11%	93.89%
‘Multi-stimulated’	M1	14,984,276	13,839,471	92.36%	96.71%
	M2	11,329,360	10,510,197	92.77%	96.32%
	M3	12,342,792	11,346,338	91.93%	96.73%

Filtered reads of the RNA libraries were de novo assembled, yielding 120,942 transcripts with an N50 of 1,736 covering 128,242,001 nucleotides. The assembled transcripts were then clustered to reduce redundancy, resulting in a final transcriptome of 93,662 transcripts covering 88,897,694 nucleotides. A BUSCO assessment of this transcriptome showed a high completeness score of 96.4%.

#### Analysis of transcriptional changes after mechanical disturbances

The RNA libraries reads mapping to the *Mimosa* transcriptome assembly ranged from 90 to 97% (Table 1). The mapping of reads to each transcript in the individual RNA libraries was then quantified. After filtering out the ‘not active’ genes (43,487 out of the 93,662 assembled transcripts were classified as ‘active’), normalization factors were assessed based on libraries size, and normalized reads counts were calculated. The MDS plot demonstrated high reproducibility, with biological replicates closely clustered and clearly distinct (Fig. S1).

**Fig. 2:**
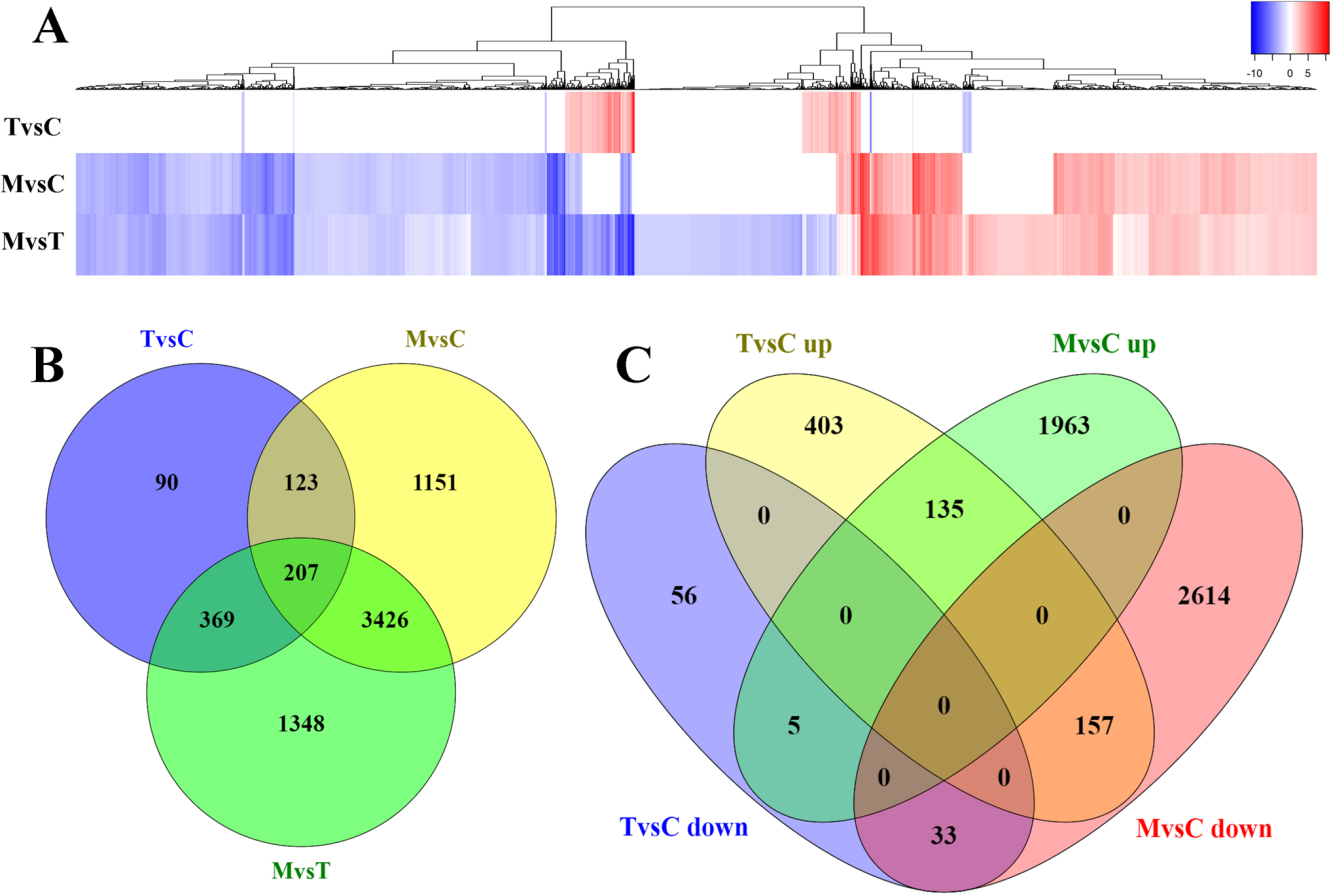
DE analyses results. **A** Heatmap reporting the LFC of each DET for the three pairwise comparisons. Color key for LFC value is displayed. **B** Venn diagram of DETs of the three comparisons ‘single-stimulated’ *vs.* ‘control’ (TvsC), ‘multi-stimulated’ *vs.* ‘control’ (MvsC), and ‘multi-stimulated’ *vs.* ‘single-stimulated’ (MvsT). **C** Venn diagram of up and down-regulated transcripts for the comparisons ‘single-stimulated’ *vs.* ‘control’ (TvsC) and ‘multi-stimulated’ *vs.* ‘control’ (MvsC)

Differential expression analysis was performed for three pairwise comparisons: plants dropped one time *vs*. control plants (‘single-stimulated’ *vs.* ‘control’), plants dropped multiple times *vs*. control plants (‘multi-stimulated’ *vs.* ‘control’), and plants dropped multiple times *vs*. plants dropped one time (‘multi-stimulated’ *vs.* ‘single-stimulated’) (Fig. 2). The DETs for the three pairwise comparisons, along with their associated analysis results, are provided in Table S1 and represented as volcano plots in Fig. S2. A summary of DET counts is presented in Table 2.

**Table 2:** DE analysis results’ summary. For the three pairwise comparisons, the numbers of down-regulated, up-regulated, and total regulated transcript were reported

Pairwise comparison	Down-regulated transcripts	Up-regulated transcripts	Total regulated transcripts
‘Single-stimulated’ *vs.* ‘control’	94	695	789
‘Multi-stimulated’ *vs.* ‘control’	2804	2103	4907
‘Multi-stimulated’ *vs.* ‘single-stimulated’	1911	3439	5350

A clustering heatmap of all the DETs was generated, revealing distinct transcript groups with varying expression patterns (Fig. 2A). In particular, this clusterization evidenced the similarities between the results of the comparisons involving plants dropped multiple times (‘multi-stimulated’ *vs.* ‘control’ and ‘multi-stimulated’ vs. ‘single-stimulated’). Single dropping shapes the expression level of 789 transcripts, among which 94 were down-regulated and 695 were up-regulated. Multiple droppings induced a differential transcriptional activity in 4907 transcripts compared to control plants, among which 2804 were down-regulated and 2103 were up-regulated. Furthermore, multiple droppings induced a differential transcriptional activity in 5350 transcripts compared to single dropped plants, among which 3439 were down-regulated and 1911 were up-regulated (Table 2). According to these numbers, single dropping induced transcriptomic changes in a smaller number of genes compared to multiple droppings, predominantly up-regulating them.

Interestingly, the number of DETs is higher in the comparison between ‘multi-stimulated’ and ‘single-stimulated’ than in the other comparisons, even surpassing the number of DETs between ‘multi-stimulated’ and ‘control’ plants. While the number of up-regulated DETs was greater than the number of down-regulated ones after a single dropping, the transcriptomic changes induced after multi-dropping consistently shifted toward the down-regulation in both ‘multi-stimulated’ *vs.* ‘control’ and ‘multi-stimulated’ *vs.* ‘single-stimulated’ comparisons. Surprisingly, this pattern was more pronounced in the ‘multi-stimulated’ *vs.* ‘single-stimulated’ comparison (Table 2), revealing a significantly larger number of expression differences than in the ‘single-stimulated’ *vs.* ‘control’ plants’ comparison.

As shown in Fig. 2B, the number of DETs shared between the comparisons involving multi-dropping was nearly ten times higher than those shared between the single-dropping and control plant comparisons (3426 versus 369, respectively). Moreover, according to the Venn diagram (Fig. 2C) of the comparisons ‘single-stimulated’ *vs.* ‘control’, and ‘multi-stimulated’ *vs.* ‘control’, most of the differentially regulated transcripts were specific for each pairwise comparison. This was evidenced also in 6-way Venn diagram in Fig. S3, where DETs were unique only in the comparisons involving ‘multi-stimulated’ samples. However, 135 and 33 transcripts that are up- or down-regulated in both comparisons were identified, respectively. Among these, transcripts with contrasting expression pattern in the two pairwise comparisons were found: 157 were up-regulated in ‘single-stimulated’ *vs.* ‘control’ and down-regulated in ‘multi-stimulated’ *vs.* ‘control’, and 5 were down-regulated in ‘single-stimulated’ *vs.* ‘control’ and up-regulated in ‘multi-stimulated’ *vs.* ‘control’.

Transcripts that resulted as differentially expressed in at least one of the three pairwise comparisons were functionally annotated. Of the 6714 DETs, a total of 5638 (84.0%), 5631 (83.9%), 4721 (70.3%), 5634 (83.9%), and 5362 (79.9%) returned at least one hit after the BLASTp analysis with the NCBI nr, RefSeq, SwissProt, TrEMBL, and Araport11 databases as subjects, respectively. The detailed functional annotation results are presented in Table S2.

### GO- and KEGG-enrichment analyses results

Enrichment analysis facilitates the classification of gene functions and the identification of over-represented biological processes and pathways, offering a comprehensive understanding of the underlying biological mechanisms. This enhances the interpretability and relevance of our transcriptomic data. Consequently, Gene Ontology (GO) and Kyoto Encyclopedia of Genes and Genomes (KEGG) terms were assigned to the entire de novo assembled *M. pudica* transcriptome to perform the required gene set enrichment analyses. Among the *M. pudica* assembled transcripts, GO terms were assigned to 14,833, while KEGG orthologs were assigned to 10,311 (Table S3). The number of enriched GO categories was 7, 14, and 16 for ‘single-stimulated’ *vs.* ‘control’, ‘multi-stimulated’ *vs.* ‘control’, and ‘multi-stimulated’ *vs.* ‘single-stimulated’ pairwise comparisons, respectively (Table 3, Fig. S3).

**Table 3:** Enriched GO categories for DETs obtained from ‘single-stimulated’ *vs.* ‘control’, ‘multi-stimulated’ *vs.* ‘control’, and ‘multi-stimulated’ *vs.* ‘single-stimulated’ pairwise comparisons

GO ID	Ontology	GO description	Adjusted *P* value	Cluster frequency	Total frequency
‘Single-stimulated’ *vs.* ‘control’					
GO:0019748	BP	secondary metabolic process	8.70E–06	24/157 15.2%	702/14881 4.7%
GO:0006519	BP	cellular amino acid and derivative metabolic process	4.06E–05	23/157 14.6%	723/14881 4.8%
GO:0019725	BP	cellular homeostasis	2.31E–02	10/157 6.3%	319/14881 2.1%
GO:0030312	CC	external encapsulating structure	3.82E–14	32/157 20.3%	537/14881 3.6%
GO:0005618	CC	cell wall	3.82E–14	32/157 20.3%	537/14881 3.6%
GO:0005773	CC	vacuole	8.06E–03	25/157 15.9%	1167/14881 7.8%
GO:0005886	CC	plasma membrane	1.96E–02	52/157 33.1%	3358/14881 22.5%
‘Multi-stimulated’ *vs.* ‘control’					
GO:0019748	BP	secondary metabolic process	5.51E–13	114/1140 10.0%	702/14881 4.7%
GO:0009628	BP	response to abiotic stimulus	1.48E–09	254/1140 22.2%	2265/14881 15.2%
GO:0006629	BP	lipid metabolic process	3.41E–08	122/1140 10.7%	923/14881 6.2%
GO:0009835	BP	ripening	1.02E–07	9/1140 0.7%	11/14881 0.0%
GO:0009607	BP	response to biotic stimulus	2.98E–06	142/1140 12.4%	1217/14881 8.1%
GO:0006950	BP	response to stress	9.35E–06	318/1140 27.8%	3270/14881 21.9%
GO:0009605	BP	response to external stimulus	2.43E–05	186/1140 16.3%	1760/14881 11.8%
GO:0005975	BP	carbohydrate metabolic process	1.42E–03	97/1140 8.5%	879/14881 5.9%
GO:0009719	BP	response to endogenous stimulus	1.42E–03	188/1140 16.4%	1918/14881 12.8%
GO:0006519	BP	cellular amino acid and derivative metabolic process	8.49E–03	78/1140 6.8%	723/14881 4.8%
GO:0005215	MF	transporter activity	1.17E–04	133/1140 11.6%	1210/14881 8.1%
GO:0016740	MF	transferase activity	1.80E–03	271/1140 23.7%	2924/14881 19.6%
GO:0003824	MF	catalytic activity	8.48E–03	581/1140 50.9%	6927/14881 46.5%
GO:0003674	MF	molecular_function	8.49E–03	897/1140 78.6%	11,153/14881 74.9%
‘Multi-stimulated’ *vs.* ‘single-stimulated’					
GO:0019748	BP	secondary metabolic process	6.07E–18	137/1303 10.5%	702/14881 4.7%
GO:0006519	BP	cellular amino acid and derivative metabolic process	2.86E–11	122/1303 9.3%	723/14881 4.8%
GO:0006629	BP	lipid metabolic process	2.23E–07	132/1303 10.1%	923/14881 6.2%
GO:0009835	BP	ripening	3.41E–07	9/1303 0.6%	11/14881 0.0%
GO:0005975	BP	carbohydrate metabolic process	2.86E–06	122/1303 9.3%	879/14881 5.9%
GO:0009628	BP	response to abiotic stimulus	3.90E–06	263/1303 20.1%	2265/14881 15.2%
GO:0009058	BP	biosynthetic process	6.48E–04	281/1303 21.5%	2609/14881 17.5%
GO:0009607	BP	response to biotic stimulus	2.92E–03	141/1303 10.8%	1217/14881 8.1%
GO:0009605	BP	response to external stimulus	1.46E–02	188/1303 14.4%	1760/14881 11.8%
GO:0030312	CC	external encapsulating structure	1.46E–02	67/1303 5.1%	537/14881 3.6%
GO:0005618	CC	cell wall	1.46E–02	67/1303 5.1%	537/14881 3.6%
GO:0005576	CC	extracellular region	3.86E–02	61/1303 4.6%	504/14881 3.3%
GO:0003824	MF	catalytic activity	3.66E–06	694/1303 53.2%	6927/14881 46.5%
GO:0016740	MF	transferase activity	6.98E–03	301/1303 23.1%	2924/14881 19.6%
GO:0005215	MF	transporter activity	2.92E–02	132/1303 10.1%	1210/14881 8.1%
GO:0003674	MF	molecular_function	4.70E–02	1013/1303 77.7%	11,153/14881 74.9%

In ‘single-stimulated’ *vs.* ‘control’ comparison, the main enriched GO categories are “cell wall” (GO:0005618) and “vacuole” (GO:0005773) for “cellular component” ontology, “secondary metabolic process” (GO:0019748), and “cellular amino acid and derivative metabolic process” (GO:0006519) for “biological process”.

**Fig. 3:**
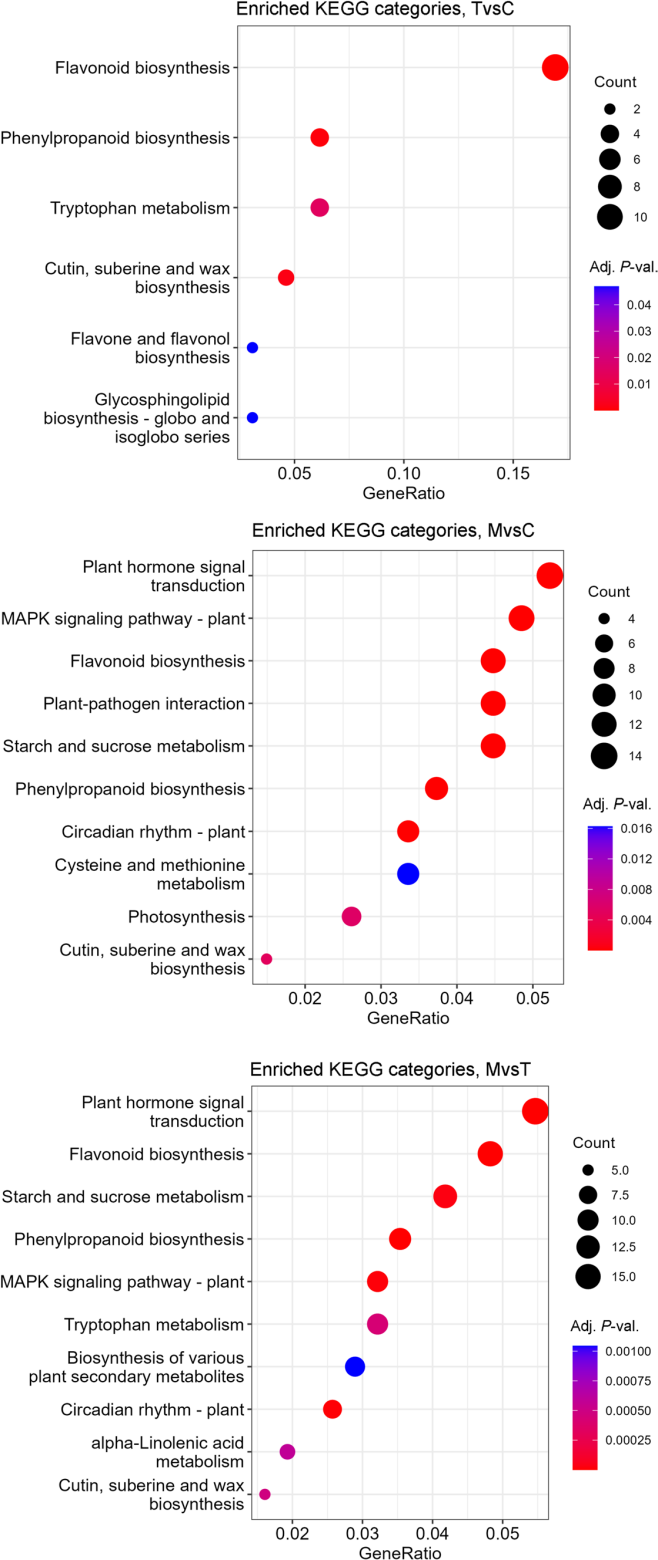
Top ten enriched KEGG pathways for ‘single-stimulated’ *vs.* ‘control’ (TvsC), ‘multi-stimulated’ *vs.* ‘control’ (MvsC), and ‘multi-stimulated’ *vs.* ‘single-stimulated’ (MvsT) pairwise comparisons. X axis represents the enrichment ratio (number of DETs belonging to the KEGG category/number of genes belonging to the same KEGG category in the background genome); Y axis represents the enriched KEGG categories ordered by the number of detected DEGs. The dots size represents the numbers of DETs included in each KEGG category. The dots color represents the adjusted *P* value for each KEGG category enrichment

On the other hand, the DETs found in the two comparisons involving the multi-dropping samples ‘multi-stimulated’ *vs.* ‘control’ and ‘multi-stimulated’ *vs.* ‘single-stimulated’ share most of the enriched GO categories (Fig. 3). In both genes subsets, “transferase activity” (GO:0016740) and “transporter activity” (GO:0005215) are the over-represented subcategories for “molecular function” ontology, while for “biological process” ontology, they share “secondary metabolic process” (GO:0019748), “cellular amino acid and derivative metabolic process” (GO:0006519), “lipid metabolic process” (GO:0006629), “ripening” (GO:0009835), “carbohydrate metabolic process” (GO:0005975), “response to abiotic stimulus” (GO:0009628), “response to biotic stimulus” (GO:0009607), and “response to external stimulus” (GO:0009605) as enriched categories. The most significantly enriched “biological process” GO categories (adjusted *P* value < 10^–7^) in the two comparisons are “lipid metabolic process” (GO:0006629), “secondary metabolic process” (GO:0019748), “ripening” (GO:0009835), and “response to abiotic stimulus” (GO:0009628). Lipid metabolism plays a crucial role in plant stress responses taking part to several defense process (Bhattacharya 2022), modulating membrane composition, producing stress-related hormones (i.e., abscisic acid), storing energy reserves, and protecting against oxidative damage. In particular, the notable transcripts linked to lipid metabolic process and highlighted in both the comparisons involving the ‘multi-stimulated’ samples were TRINITY_DN3421_C0_G1_I1, TRINITY_DN3421_C0_G1_I2, TRINITY_DN3421_C0_G1_I3, annotated as Alpha-dioxygenase 2, which is described to be involved in the oxidative degradation of fatty acids and in the formation of oxylipins (Blée 2002), implicated in signaling and stress response. TRINITY_DN2840_C0_G1_I1, TRINITY_DN2840_C0_G1_I2, is annotated as the protein 3-like responsible to the elongation of fatty acids; TRINITY_DN414_C0_G1_I3, annotated as lysophospholipid acyltransferase LPEAT2 (Jasieniecka-Gazarkiewicz et al. 2016), a protein involved in the restructuring of phospholipids and in the regulation of the fluidity and functionality of cell membranes; TRINITY_DN4194_C0_G1_I1, annotated as a chloroplastic isoform of omega-6 fatty acid desaturase, which catalyzes the introduction of double bonds into fatty acids, promoting the synthesis of polyunsaturated fatty acids essential for membrane structure and lipid signaling; and finally, TRINITY_DN455_C0_G2_I1, annotated as 3-ketoacyl-CoA synthase 1 (Luo et al. 2024), a key enzyme in the biosynthesis of very long-chain fatty acids, important for cuticular waxes and structural lipids.

“Biological process” GO categories “response to stress” (GO:0006950) and “response to endogenous stimulus” (GO:0009719) are enriched specifically in ‘multi-stimulated’ *vs.* ‘control’ DETs, while “biosynthetic process” (GO:0009058) is specifically enriched in ‘multi-stimulated’ *vs.* ‘single-stimulated’ comparison (Fig. 3). This DETs’ subset also presents over-represented “external encapsulating structure” (GO:0030312) and “cell wall” (GO:0005618) “cellular component” GO categories.

Regarding the KEGG pathway enrichment analysis, the number of enriched KEGG pathways was 6, 17, and 30 for ‘single-stimulated’ *vs.* ‘control’, ‘multi-stimulated’ *vs.* ‘control’, and ‘multi-stimulated’ *vs.* ‘single-stimulated’ pairwise comparisons, respectively (Table S4), and the top 10 enriched categories for each comparison are shown in Fig. 3. As evidenced, there were fewer enriched KEGG pathways with the identified DETs when comparing’stimulated’ *vs.* ‘control’ than when comparing ‘multi-stimulated’ *vs.* ‘control’ and ‘multi-stimulated’ *vs.* ‘single-stimulated’. Interestingly, the “flavonoid biosynthesis pathway” category is significantly enriched, reaching a GeneRatio > 0.005 (number of DETs belonging to the KEGG category/number of genes belonging to the same KEGG category in the background genome). Contrarily, the DETs found in ‘multi-stimulated’ *vs.* ‘control’ and ‘multi-stimulated’ *vs.* ‘single-stimulated’ comparisons originated a greater number of enriched KEGG pathways, most of which are shared between the two subsets. In particular, “plant hormone signal transduction” (map04075), “polypropanoid biosynthesis” (map00940), and “circadian rhythm” (map04712) are among the most significantly enriched KEGG metabolic pathways in both the comparisons. The KEGG “flavonoid metabolism” (map00941) category, which includes phenylpropanoid, flavone and flavonol, and isoflavonoid and anthocyanin biosynthetic pathways, was enriched in all three comparisons, evidencing the fundamental role of flavonoids in plant response to both single and multiple stresses.

Furthermore, the “plant hormone signal transduction” (map04075) category was significantly enriched (adjusted *P* value < 0.05) in both ‘multi-stimulated’ *vs.* ‘control’ and ‘multi-stimulated’ *vs.* ‘single-stimulated’ comparisons, highlighting the role of multiple stress on hormones metabolic networks. In particular, map04075 includes the biosynthesis pathways of zeatin, which contribute to cytokinin biosynthesis; diterpenoid pathways, which contribute to gibberellin biosynthesis; and cysteine and methionine biosynthesis, which contribute to the ethylene biosynthetic pathway. It also includes brassinosteroid biosynthesis, jasmonic acid biosynthesis, and tryptophan metabolism, which contribute to the biosynthesis pathways of auxin and abscisic acid.

### Characterization of genes with different transcriptional patterns

**Fig. 4:**
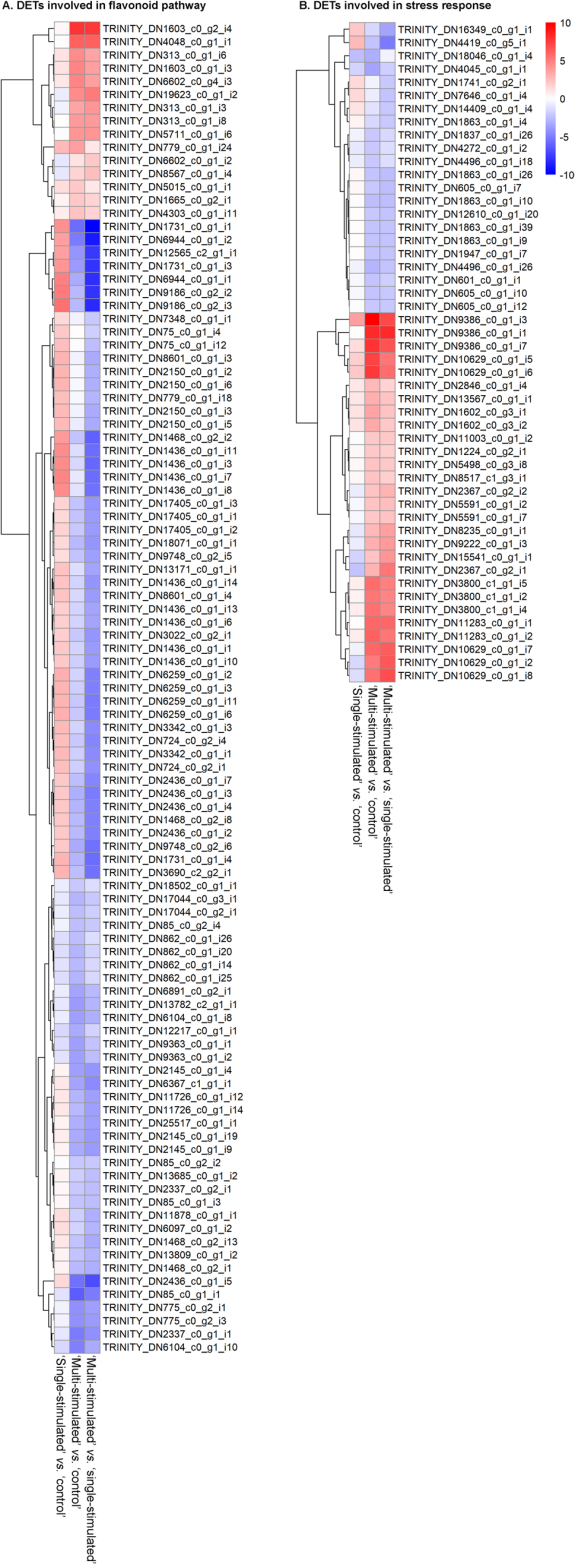
Heatmaps visualizing the log2 (fold change) values of the DETs involved in flavonoids pathways (**A**) and biotic and abiotic stress response (**B**) for ‘single-stimulated’ *vs.* ‘control’, ‘multi-stimulated’ *vs.* ‘control’ and ‘multi-stimulated’ *vs.* ‘single-stimulated’ pairwise comparisons. Color key is displayed

The expression level of several genes showed a similar trend in the comparisons involving plants stimulated multiple times (‘multi-stimulated’ *vs.* ‘single-stimulated’ and ‘multi-stimulated’ *vs.* ‘control’), and this trend was different from that observed when comparing’stimulated’ *vs.* ‘control’ (Fig. 4). 1405 out of 6714 DETs showed an up-regulation in ‘multi-stimulated’ *vs.* ‘single-stimulated’ and ‘multi-stimulated’ *vs.* ‘control’ comparisons, while they were not regulated in the’stimulated’ *vs.* ‘control’ comparison. Among these, TRINITY_DN1556_c0_g1_i1 (LFC = 0,28; 7,22; 6,95, for’stimulated’ *vs.* ‘control’, ‘multi-stimulated’ *vs.* ‘control’ and ‘multi-stimulated’ *vs.* ‘single-stimulated’, respectively) and TRINITY_DN1556_c0_g1_i5 (LFC = − 0,25; 7,15; 7,40 for’stimulated’ *vs.* ‘control’, ‘multi-stimulated’ *vs.* ‘control’ and ‘multi-stimulated’ *vs.* ‘single-stimulated’, respectively), both showing a high divergence in expression regulation have been classified as participating in the GO categories “response to stress”, “response to abiotic stimulus”, “carbohydrate metabolic process”, and “biosynthetic process “. The latter category was significantly enriched only in the ‘multi-stimulated’ *vs.* ‘single-stimulated’ comparison (Table S4). In addition, an evident difference in gene expression among’stimulated’ *vs.* ‘control’ with respect to ‘multi-stimulated’ *vs.* ‘single-stimulated’ and ‘multi-stimulated’ *vs.* ‘control’ comparisons was observed for TRINITY_DN21409_c1_g2_i1, (LFC = − 1,13; 4,70; 5,84; for’stimulated’ *vs.* ‘control’, ‘multi-stimulated’ *vs.* ‘control’ and ‘multi-stimulated’ *vs.* ‘single-stimulated’, respectively) included in “response to abiotic stimulus” and “response to stress”. Only 5 DETs were characterized by a down-regulation in’stimulated’ *vs.* ‘control’ comparison and an up-regulation in the other two comparisons. In 157 DETs, an opposite trend was observed, with an up-regulation of gene expression in the’stimulated’ *vs.* ‘control’ comparison and a down-regulation in the other two comparisons. Interestingly, some of these genes belong to “phenylpropanoid biosynthesis” and “flavonoids biosynthesis” (Fig. 4A). Some of the DETs involved in these pathways are often assigned as chalcone-flavanone isomerase family protein (as TRINITY_DN2436_c0_g1_i2, TRINITY_DN2436_c0_g1_i4 and TRINITY_DN2436_c0_g1_i7).

Among the up-regulated genes in ‘single-stimulated’ *vs.* ‘control’ comparison and down-regulated in the other two comparisons (‘multi-stimulated’ *vs.* ‘control’ and ‘multi-stimulated’ *vs.* ‘single-stimulated’) involved in “flavonoids biosynthesis”, there were TRINITY_DN11871_c0_g1_i1, TRINITY_DN12460_c0_g1_i1, TRINITY_DN12460_c0_g1_i2., TRINITY_DN12716_c0_g2_i1, TRINITY_DN13047_c0_g1_i1, TRINITY_DN14004_c0_g1_i1, TRINITY_DN17981_c1_g1_i4, TRINITY_DN17981_c1_g1_i5, TRINITY_DN21242_c0_g1_i1 TRINITY_DN2154_c0_g1_i1 TRINITY_DN2285_c0_g1_i2 and TRINITY_DN8071_c0_g1_i8. Interestingly, four of these (TRINITY_DN10306_c0_g1_i1, TRINITY_DN10306_c0_g1_i3, TRINITY_DN10306_c0_g1_i2, TRINITY_DN10306_c0_g1_i5) were annotated as leucoanthocyanidin dioxygenase, involved in proanthocyanins, class of polyphenols with various pharmacological properties, biosynthesis.

In the observed cases, the LFC in the’stimulated’ *vs.* ‘control’ comparison was completely opposite to the LFC in the ‘multi-stimulated’ *vs.* ‘single-stimulated’ comparison, indicating that the genes respond significantly differently to the intensity and frequency of the stimulation.

There were no genes down-regulated in both’stimulated’ *vs.* ‘control’ and ‘multi-stimulated’ *vs.* ‘control’ comparisons and up-regulated in ‘multi-stimulated’ *vs.* ‘single-stimulated’, but 441 transcripts were up-regulated only in the comparison ‘multi-stimulated’ *vs.* ‘single-stimulated’ and not regulated in the other comparisons, and mostly involved in KEGG pathways referred to the biological process of “plant hormone signals transduction” (map04075) (i.e., TRINITY_DN48442_c0_g1_i1, TRINITY_DN7301_c0_g1_i3, and TRINITY_DN7339_c2_g1_i1) and “phenylpropanoid biosynthesis” (map00940) (i.e., TRINITY_DN4232_c0_g1_i6).

Interestingly, 32 genes were down-regulated in all the three comparisons, indicating a broad effect of mechanical stimulation on gene expression. This pattern was observed in both multiple or single stress conditions compared to control plants, as well as in multiple *vs.* single-stimulated plants. Most were identified as hypothetical or unidentified proteins, but it seems that the diffused down-regulation in all the three comparisons was frequent in transcriptional regulators (EXS and WRKY family), enzymes, and disease resistance genes. Conversely, only 28 genes were up-regulated in all the comparisons, explaining in the same manner the effect of mechanical perturbation on unstressed or less-stressed plants. In this case, the involved genes determined the overexpression of DNA-binding proteins as zinc fingers and helix–loop–helix, stress-induced proteins, transferases, and iron transporters.

In general, the most differentially regulated gene categories in the three comparisons were: (1) among transcription factors (TFs), NACs, WRKYs, UNE12-like, MYB108, MYB6 bZIPs, RAX2, MTERF15, MTERF2, ERF113-like, AP2/ERF, heat shock proteins, and MYB6 which are usually up-regulated in ‘multi-stimulated’ *vs.* ‘control’ and ‘multi-stimulated’ *vs.* ‘single-stimulated’ comparisons; they were not subjected to variation in ‘single-stimulated’ *vs.* ‘control’ comparison; (2) several flavonoid biosynthesis-related genes, such as flavone synthase of cytochrome P450 family, dihydroflavonol-4-reductase, 2-hydroxyisoflavanone dehydratase, anthocyanidin reductase, and flavanone 3-hydroxylase, were mainly up-regulated in ‘single-stimulated’ *vs.* ‘control’ comparison and down or not differentially regulated in the comparisons involving multi-dropped plants (Fig. 4A); (3) among stress-related genes related to plant’ stress response, both biotic and abiotic, universal stress-related proteins, early responsive to dehydration 15 proteins, NST1-like proteins, heat stress, and heat shock proteins were found to be mostly regulated in the comparisons which include the multiple stimulation to plants and did not show consistent variation in ‘single-stimulated’ *vs.* ‘control’ comparison (Fig. 4B).

## Discussion

*Mimosa pudica* is a plant known for its capability of folding leaves in response to biotic contacts and abiotic disturbances (Bakshi et al. 2023). Thigmonasty is a type of plant movement in which rapid leaf or leaflet closure occurs in response to physical touch or mechanical stimuli, as observed in carnivorous plants like *Dionaea muscipula* after insect stimulation (Braam 2005), and in *M. pudica* (Volkov et al. 2010a; Monshausen and Haswell 2013). The molecular and cellular electrical mechanisms that regulate leaf movements are the subject of recent studies (Stolarz and Trębacz 2021). Nevertheless, the genetic basis of these regulations is poorly understood, in particular, when discriminating type, intensity, and frequency of the perceived stimulation (Volkov et al. 2010b; Hagihara and Toyota 2020). Furthermore, while several genes involved in multiple stress responses caused by different stressor conditions (salt, drought, and temperature) have been largely investigated (Shao et al. 2015; Tan et al. 2023), only few data on the differential activation of genes in plants subjected to single or repetitive mechanical stimulation are available (Pan et al. 2012; Sewelam et al. 2014; Sato et al. 2024). In many cases, plants respond to abiotic and biotic stresses activating defensive pathways that can ‘prepare’ plants to respond more effectively to similar future stresses, resulting in quicker, stronger, or more persistent reactions. It is unclear whether this also applies to mechanical stresses, and in particular to the mechanism involved in signal perception and transduction that lead to the leaf folding behavior of *M. pudica* (Michmizos and Hilioti 2019).

We show that the gene regulatory network determining *M. pudica* leaf response after multiple mechanical stress was different with respect to both unstressed plants and plants subjected to a single stress event. First, at phenotypic level, the chlorophyll fluorescence of the plants analyzed, a widely used method to test the activity of PSII (therefore, the photochemical efficiency) under abiotic and biotic stresses. Reduction of the maximum quantum yield of PSII in dark-adapted leaves (*F*_v_/*F*_m_) indicates the presence of stress affecting photochemical efficiency, whereas the quantum yield of PSII in illuminated leaves is a proxy of photosynthesis and photorespiration, and of photosynthetic electron transport (Maxwell and Johnson 2000). Our measurements suggest that the maximal photochemical efficiency is not impaired by either single or repeated mechanical stress, as *F*_v_/*F*_m_ remained high in all treatments. However, the lower quantum yield of PSII in leaves of ‘single-stimulated’ and ‘multi-stimulated’ plants under illumination, compared to ‘control’ plants, indicates a significant inhibition of photosynthesis and/or photorespiration. From these data, we cannot say whether the inhibition of photosynthesis was caused by any of the three classic photosynthetic limitations: diffusive, biochemical, or photochemical (Flexas et al. 2004). The latter might seem less likely, given that *F*_v_/*F*_m_ was similar in all dark-adapted samples. Nevertheless, ATP as a source of energy could be a significant factor influencing photochemistry of illuminated plants, limiting regeneration of ribulose bisphosphate and therefore photosynthesis (Williams and Bennett 1982). ATP is crucial for sustaining various cellular functions, including leaf movement (Williams and Bennett 1982). H^+−^ATPase is instrumental in activating membrane hyperpolarization that causes guard cells swelling under blue light (Inoue et al. 2010). The *M. pudica* leaf folding is controlled by the available energy, being slowed down or stopped under dim light or in the dark (Roblin et al. 2024). Plasmodesmata are rich of ATP and the pulvinus of *M. pudica*, where leaf movement is activated, are rich of plasmodesmata. However, H^+−^ATPase was not found in plasmodesmata of *M. pudica* pulvinus, which may indicate different regulation of the ATP content in these structures (Fleurat-Lessard et al. 1995). Although the mechanisms regulating ATP levels in pulvini are not yet fully understood, ATP could potentially play a role as an energy source limiting both *M. pudica* leaf folding in response to repeated mechanical stimulation and photosynthetic electron transport. However, this remains a speculative hypothesis, and future studies involving direct measurements or accurate estimations of ATP content will be necessary to validate this possibility. Several genes involved in responses to stress may also play a role in regulating ATP production and utilization. For instance, genes that respond to salt, drought, and temperature stresses have been shown to influence metabolic pathways, including those generating ATP, while also regulating the photosynthetic efficiency (Krasensky and Jonak 2012; Shelake et al. 2022).

In line with our early sampling strategy, designed to capture the immediate molecular responses of *Mimosa pudica*, our findings highlighted a large number of DETs potentially involved in the early signaling events underlying the plant’s rapid movements in all the comparisons among stress conditions. A greater number of DETs were observed in the comparison between ‘multi-stimulated’ *vs.* ‘single-stimulated’ plants compared to the DETs of ‘multi-stimulated’ or ‘single-stimulated’ *vs.* ‘control’, suggesting a progressive activation of metabolic pathways under repeated mechanical disturbance.

In the ‘single-stimulated’ *vs.* ‘control’ comparison, only 6 up-regulated DETs had a LFC > 9, indicating genes over-expressed in the single-dropping condition. In particular, 3 of these belong to the “flavonoid biosynthesis” (KEGG map00941): TRINITY_DN17981_c1_g1_i5, a protein belonging to the O-fucosyltransferase family, TRINITY_DN13047_c0_g1_i1, a bifunctional 3-dehydroquinate dehydratase/shikimate dehydrogenase-like protein involved in the biosynthesis of aromatic amino acids from the metabolism of carbohydrates, and TRINITY_DN2285_c0_g1_i3, a leucoanthocyanidin reductase. However, these genes were not further up-regulated in ‘multi-stimulated’ plants.

Flavonoids, highly enriched in differentially expressed genes, are key phenylpropanoids like anthocyanins, flavonols, and proanthocyanidins, essential for plant growth, defense, and stress responses. They enhance resilience to drought and salt stress by activating SOD and POD enzymes to scavenge ROS, while also regulating stomatal aperture via abscisic acid, impacting photosynthesis and transpiration (Wang et al. 2021). Besides these, brassinosteroids (Planas-Riverola et al. 2019), enhanced oxidative stress resistance, and tryptophan-derived compounds are able to mediate stress signaling, protecting the plant ensuring its survival under adverse conditions (Corpas et al. 2021).

The flavonoid genes up-regulated after a single mechanical stress are specifically annotated as involved in the synthesis of (i) coumarate ligase (TRINITY_DN2181_c0_g1_i1, TRINITY_DN2181_c0_g1_i2), which plays a key role in lignin biosynthesis and the diversification of flavonoids and phenolics essential for plant growth and defense (Wang et al. 2016); (ii) flavanone hydroxylase (TRINITY_DN6259_c0_g1_i3, TRINITY_DN6259_c0_g1_i6, TRINITY_DN3690_c2_g2_i), and (iii) flavonol synthase (TRINITY_DN3342_c0_g1_i3, TRINITY_DN3342_c0_g1_i1, TRINITY_DN724_c0_g2_i4, TRINITY_DN724_c0_g2_i1), key enzymes for flavonoid biosynthetic pathway; (iv) leucoanthocyanidin reductase (TRINITY_DN2285_c0_g1_i2, TRINITY_DN2285_c0_g1_i1, TRINITY_DN6944_c0_g1_i3), which catalyzes the reduction of leucoanthocyanins into anthocyanins and other flavonoids, crucial for plant coloration and defense; (v) chalcone synthase (TRINITY_DN3023_c0_g2_i1, TRINITY_DN1019_c0_g1_i2, TRINITY_DN2436_c0_g1_i7, TRINITY_DN13661_c0_g1_i1), catalyzes the first step in flavonoid biosynthesis, producing chalcones, essential intermediates for various flavonoids. Phenylpropanoids are rapidly regulated under stress, with polyphenols peaking during the hottest hours, while simpler antioxidants are produced at other times of the day (Tattini et al. 2015). Our results indicate that flavonoid biosynthesis is triggered early by a single stress but not enhanced by repeated stress. Under multiple stresses, plants may shift energy from costly defenses like flavonoids to other survival pathways (Simms and Triplet 1994; Pissolato et al. 2024). In our specific case, ‘multi-stimulated’ plants may have shifted carbon and energy from flavonoid production to other pathways critical for long-term survival. Consistently, the genes involved in response to stress (biotic, abiotic, and external stimuli, according to the enriched GO categories GO:0009628, GO:0009607, and GO:0009605) were mainly up-regulated in ‘multi-stimulated’ plants.

Accordingly, repeated stress can lead to desensitization of the classical plant’s signaling systems, reducing activation of the genes involved in flavonoid pathway. At the same time, we observed the up-regulation of several transcriptional factors (TFs), including members of the WRKY, MYB, AP2/ERF, and heat shock transcription factor (HSF) families (Meraj et al. 2020). These TFs are known to play central roles in regulating plant responses to biotic and abiotic stresses. For instance, WRKYs are involved in stress memory and defense priming, MYBs regulate secondary metabolism and several stress adaptations, AP2/ERFs mediate responses to ethylene and abiotic signals, and HSFs are crucial in heat and oxidative stress responses (Joshi et al. 2016; Ma et al. 2022). Their increased expression following repeated stimulation may reflect a transcriptional reprogramming that enhances defense capacity, supports adaptive plasticity, and potentially contributes to memory-like responses. Further inspection of the ‘multi-stimulated’ *vs.* ‘single-stimulated’ results showed the up-regulation of 441 transcripts that are not responding to the single stress event. These genes are mostly involved in hormone signals transduction, and some of these also in the phenylpropanoid biosynthesis, confirming the role of these molecules in mediating stress responses (Takahashi et al. 2019). This result may indeed indicate that hormonal activation may occur as a second step in the sequence, leading to the adaptation to stress (absence of leaf movement, in the case of *Mimosa*).

Some up-regulated genes may be crucial for memory-related processes involved in the response to repetitive stress. Recently (Pratx et al. 2024), the role of transcriptional memory in response to acclimation to abiotic or biotic stresses was explored, focusing on the differences among reception of a single stimulus (Type I) or of recurrent stimuli (Type II). Dynamic changes in TFs regulation and chromatin accessibility were identified as fundamental to trigger stress mnemonic mechanism (Pratx et al. 2024). We also observed an up-regulation of genes related to ‘lipid metabolic processes’ and ‘carbohydrate metabolic processes’ (Fig. 3), which may suggest the possible activation of more complex and structural metabolic pathways potentially involved in managing long-term stress responses or memory-like processes.

The leaf folding response of *Mimosa pudica* to mechanical disturbances highlights its significance as a model organism for studying leaf responses under mechanical stress in plants. Our investigation into the gene regulatory networks involved in stress-induced leaf closure uncovered many transcriptomic differences between single and multiple stress events. Notably, most identified DETs are associated with flavonoid biosynthesis, particularly when plants were subjected to a single stress event, while pathways related to abiotic stress responses were mainly regulated as a form of long-term stress response following repetitive stimulation. These findings not only enhance our understanding of the molecular mechanisms underlying plant responses to mechanical stress but also provide valuable insights into the adaptive strategies employed by *M. pudica* in fluctuating environments. We did not consider additional lines, as the data presented provide consistent and sufficient pieces of evidence. Nevertheless, to gain a more comprehensive understanding of the mechanically induced changes in plant behavior or physiology, the transcript levels in the pulvinus should have been measured or should be considered in future studies. All together, these knowledge paves the way for potential genetic improvement approaches by identifying candidate genes and pathways that could be targeted to enhance long-term or multi-stress resilience in plants. Understanding the molecular mechanisms underlying these processes opens avenues for various stress management strategies, including biotechnological interventions such as genetic modifications or selective breeding, to develop crops with improved responses to mechanical stimuli, better adaptation to environmental challenges, and enhanced survival strategies in changing ecosystems.

## Supplementary Information

Below is the link to the electronic supplementary material.

Supplementary file1 Table S1 DETs analysis results for ‘single-stimulated’ vs. ‘control’, ‘multi-stimulated’ vs. ‘control’ and ‘multi-stimulated’ vs. ‘single-stimulated’ pairwise comparisons. For each DET, the log2(fold change), the log2(counts per million), the Likelihood Ratio, the P-value and the False Discovery Rate values were reported (XLSX 992 KB)

Supplementary file2 Table S2 DETs functional annotation. For each subject database used with blastx-fast analysis, the best hit, its description and the e-value were reported (XLSX 1128 KB)

Supplementary file3 Table S3 GO and KEGG terms assigned to the M. pudica assembled transcriptome. The transcripts with no assigned terms were not reported (XLSX 1103 KB)

Supplementary file4 Table S4 Enriched KEGG pathways for DETs obtained from’stimulated’ vs. ‘control’, ‘multi-stimulated’ vs. ‘control’, and ‘multi-stimulated’ vs. ‘single-stimulated’ pairwise comparisons. Each record reports the KEGG pathway ID, its description, the ratio of genes in the pathway to the total number of genes (GeneRatio), the ratio of genes in the pathway to the total number of background genes (BgRatio), enrichment P-value, adjusted P-value, q-value, and the orthologues associated with the pathway (XLSX 17 KB)

Supplementary file5 Fig. S1 Multidimensional scaling (MDS) plot for the eight RNA libraries. Blue, green and red triangles represent ‘control’, ‘single-stimulated’ and ‘multi-stimulated’ plants, respectively (TIF 41 KB)

Supplementary file6 Fig. S2 Volcano plots of log2(fold change) versus -log10(false discovery rate) resulting from the differential expression analyses for the comparisons ‘single-stimulated’ vs. ‘control’ (TvsC), ‘multi-stimulated’ vs. ‘control’ (MvsC) and ‘multi-stimulated’ vs. ‘single-stimulated’ plants (MvsT). Differentially expressed transcripts (FDR < 0.05; |LFC|> 2) were represented with red dots (TIF 1591 KB)

Supplementary file7 Fig. S3 Venn diagram of up- and down-regulated transcripts for the comparisons ‘single-stimulated’ vs. ‘control’ (TvsC), ‘multi-stimulated’ vs. ‘control’ (MvsC) and ‘multi-stimulated’ vs. ‘single-stimulated’ plants (MvsT) (TIF 706 KB)

Supplementary file8 Fig. S4 Gene ontology (GO) enrichment analysis results for DETs obtained from ‘single-stimulated’ vs. ‘control’, ‘multi-stimulated’ vs. ‘control’ and ‘multi-stimulated’ vs. ‘single-stimulated’ pairwise comparisons. The circles are shaded based on significance level, and their radius is proportional to the number of DETs included in the corresponding GO category. Color key for adjusted P-value is displayed (TIF 1186 KB)

## Data Availability

RNA-Seq raw reads data have been deposited and made available at EMBL-EBI under the accession number E-MTAB-14230.

## Abbreviations

DET: Differential expressed transcript
LFC: Log2 fold change
