# Supplementary figures and images for: The transcriptional mechanism behind Mimosa pudica leaf folding in response to mechanical disturbance
Source: Planta. 2025 Oct 6;262(5):114. doi: 10.1007/s00425-025-04830-x (PMC12500843; doi:10.1007/s00425-025-04830-x)

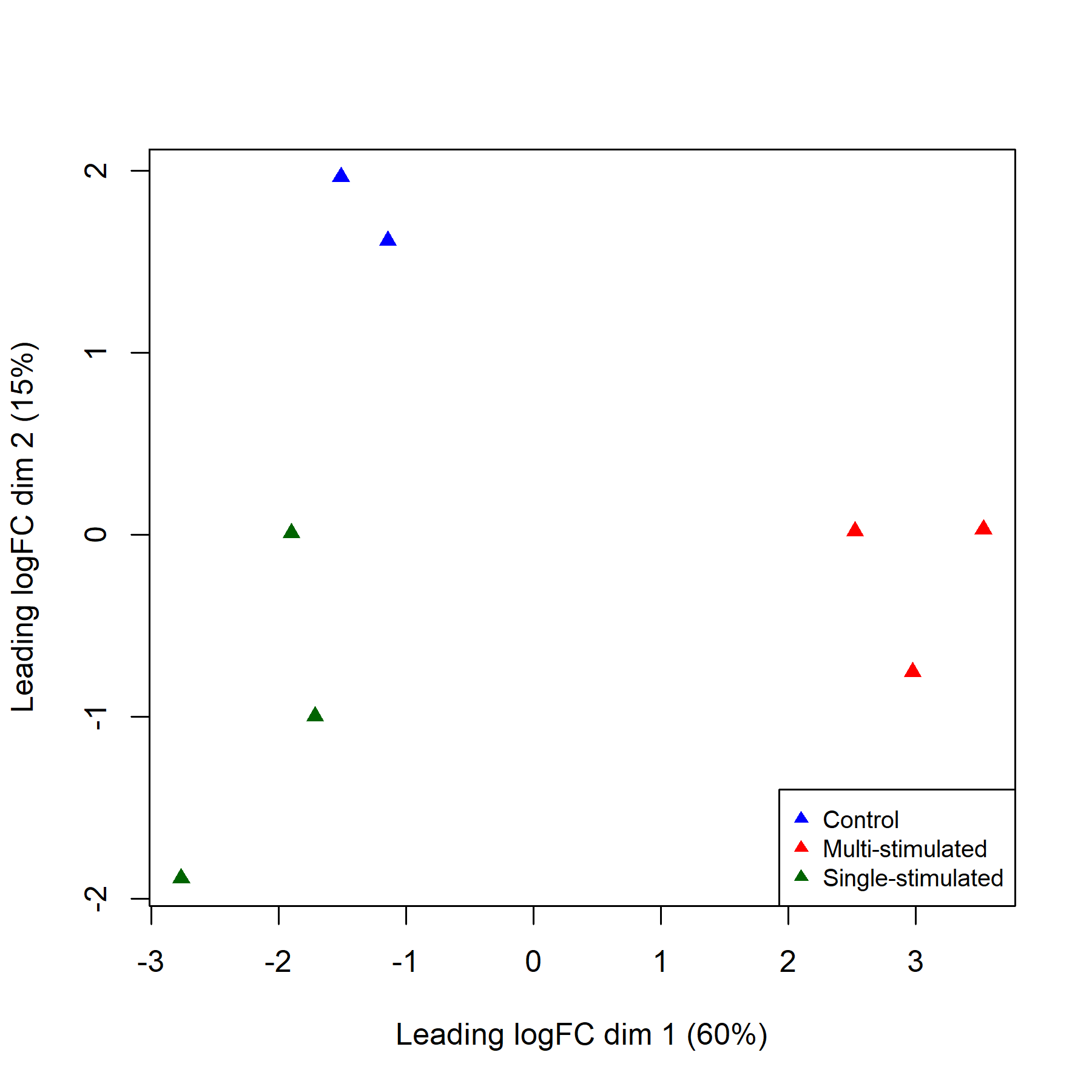

Supplement: Supplementary file 5 — Supplementary file5 Fig. S1 Multidimensional scaling (MDS) plot for the eight RNA libraries. Blue, green and red triangles represent ‘control’, ‘single-stimulated’ and ‘multi-stimulated’ plants, respectively (TIF 41 KB) [file 425_2025_4830_MOESM5_ESM.tif]

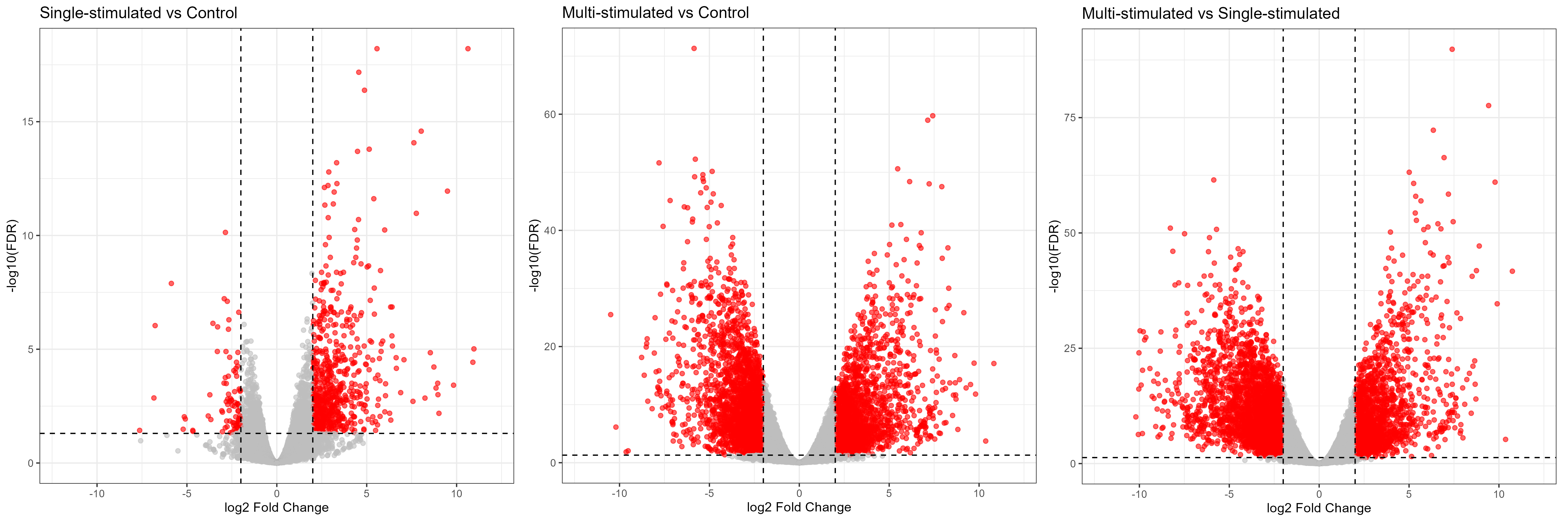

Supplement: Supplementary file 6 — Supplementary file6 Fig. S2 Volcano plots of log2(fold change) versus -log10(false discovery rate) resulting from the differential expression analyses for the comparisons ‘single-stimulated’ vs. ‘control’ (TvsC), ‘multi-stimulated’ vs. ‘control’ (MvsC) and ‘multi-stimulated’ vs. ‘single-stimulated’ plants (MvsT). Differentially expressed transcripts (FDR < 0.05; |LFC|> 2) were represented with red dots (TIF 1591 KB) [file 425_2025_4830_MOESM6_ESM.tif]

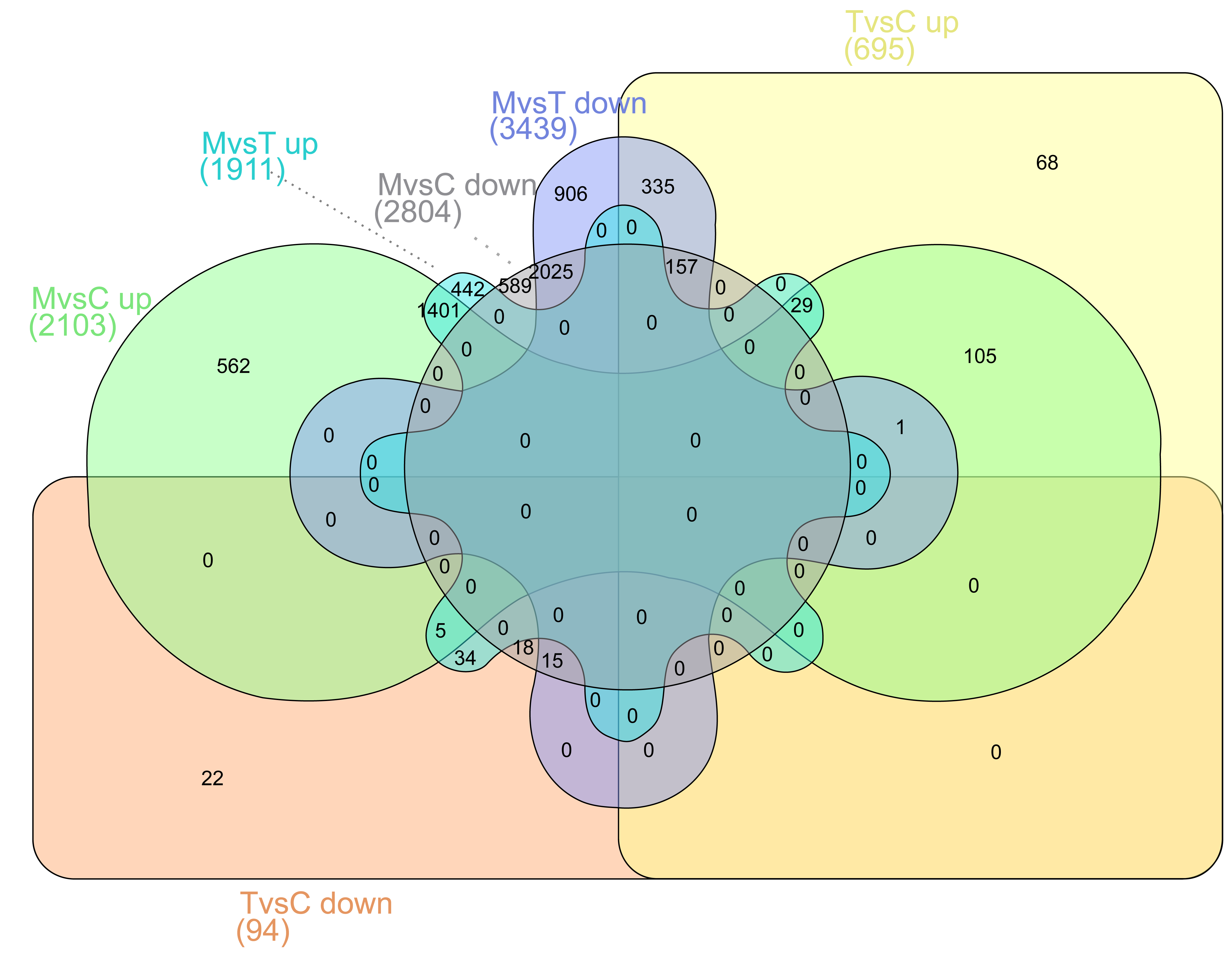

Supplement: Supplementary file 7 — Supplementary file7 Fig. S3 Venn diagram of up- and down-regulated transcripts for the comparisons ‘single-stimulated’ vs. ‘control’ (TvsC), ‘multi-stimulated’ vs. ‘control’ (MvsC) and ‘multi-stimulated’ vs. ‘single-stimulated’ plants (MvsT) (TIF 706 KB) [file 425_2025_4830_MOESM7_ESM.tif]

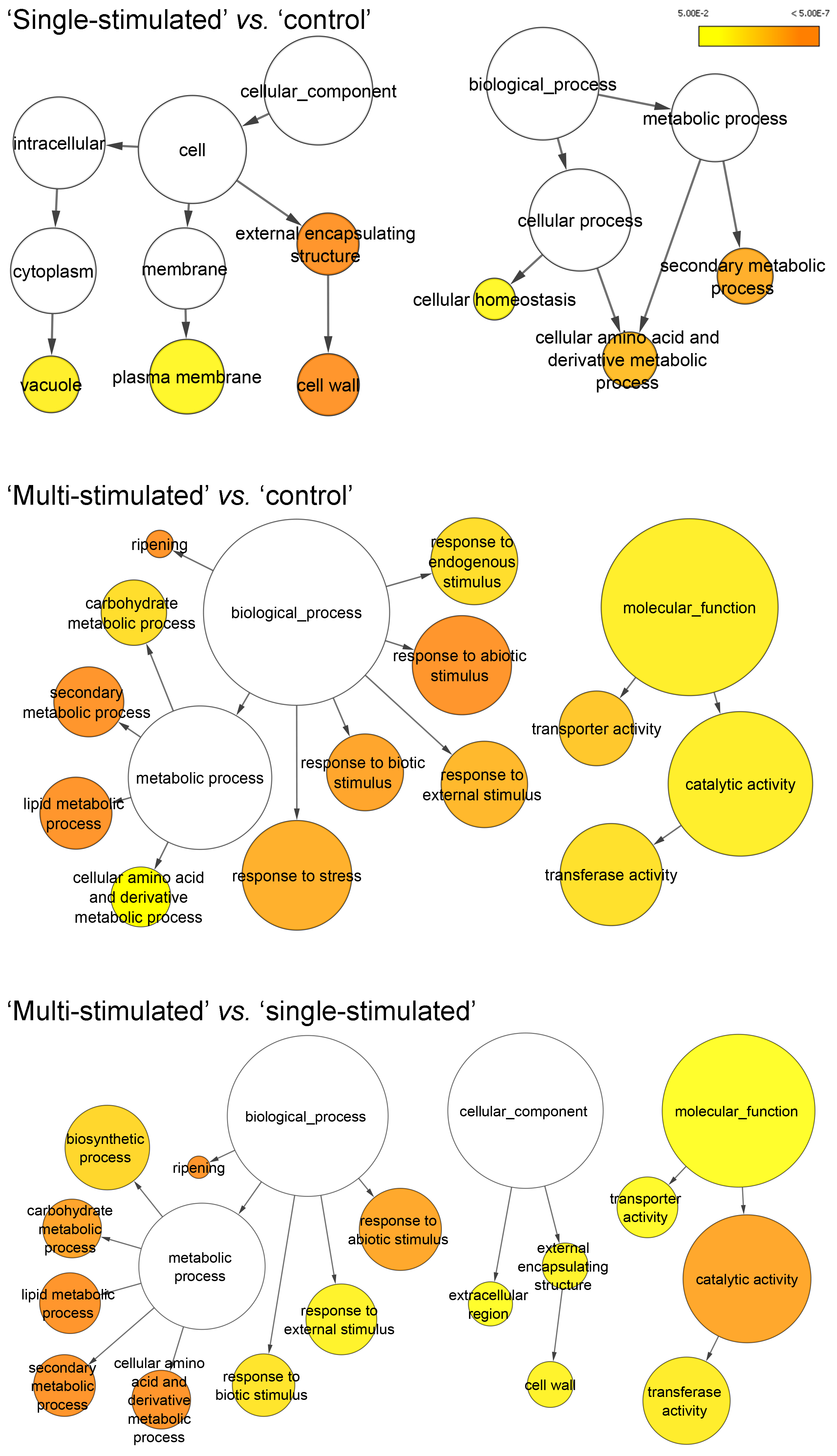

Supplement: Supplementary file 8 — Supplementary file8 Fig. S4 Gene ontology (GO) enrichment analysis results for DETs obtained from ‘single-stimulated’ vs. ‘control’, ‘multi-stimulated’ vs. ‘control’ and ‘multi-stimulated’ vs. ‘single-stimulated’ pairwise comparisons. The circles are shaded based on significance level, and their radius is proportional to the number of DETs included in the corresponding GO category. Color key for adjusted P-value is displayed (TIF 1186 KB) [file 425_2025_4830_MOESM8_ESM.tif]
